# Nonclinical Safety Pharmacology Study of the Herbal Product HAD-B1

**DOI:** 10.1155/2021/2162986

**Published:** 2021-11-09

**Authors:** Soo-Dam Kim, Jae-Ho Yang, Eun-Bin Kwag, Ji-Hye Park, So-Jung Park, Hwa-Seung Yoo

**Affiliations:** ^1^East West Cancer Center, Daejeon Korean Medicine Hospital, Daejeon University, 75, 176 Bun-Gil, Daedeok-Daero, Seo-Gu, Daejeon 35-235, Republic of Korea; ^2^East West Cancer Center, Seoul Korean Medicine Hospital, Daejeon University, 32, Beobwon-Ro 11-Gil, Songpa-Gu, Seoul 05-836, Republic of Korea

## Abstract

HAD-B1 is a Korean herbal formula designed to treat solid tumors, and through cell experiments, it has proven to have an anticancer effect. The current study aims to test the safety of HAD-B1. This experiment is under the regulation of ICH. In order to find if HAD-B1 has any effect on the CNS, 0, 500, 1000, and 2000 mg/kg/day of HAD-B1 were orally administered to male and female rats once. To discover any effect on the respiratory system, 0, 500, 1000, and 2000 mg/kg/day of HAD-B1 were orally given to male rats followed by measuring the respiratory rate, tidal volume, and minute respiratory volume. To assess the possibility of a delayed QT period as a result of the drug administration, hERG analysis was conducted at 0, 0.1, 0.3, and 1 *μ*g/ml. To assess any effect on the cardiovascular system, 0, 500, 1000, and 2000 mg/kg/day of HAD-B1 were orally given to male beagle dogs once followed by temperature, blood pressure, ECG, and heart rate analyses. There were no clinically significant changes in both male and female rats on assessing any effects on the CNS. There were no clinically significant changes in male rats' respiratory assessment. There were no clinically significant changes in hERG analysis results. There were no clinically significant changes in the cardiovascular system of male beagle dogs. Our results demonstrate that HAD-B1 is a safe herbal formula that does not have a clinically significant effect on the CNS, respiratory, and cardiovascular systems.

## 1. Introduction

Cancer is one of the global leading causes of death and ranks 1st or 2nd in many countries around the world [1]. Among all cancer, lung cancer is the highest incidence of cancer and is the first cause of cancer-related death [1, 2]. Since the development of lung cancer is strongly related to smoking, the incidence and mortality rates are gradually decreasing in countries that have successfully implemented smoking cessation campaigns but are still increasing in developing countries with high smoking rates [3]. In Korea, the 5-year relative survival rate for lung cancer patients was 27.1%, the second lowest after pancreatic cancer (11.4%) [4]. According to statistics in the U.S. in 2019, the 5-year relative survival rate for lung cancer patients was 19%, with pancreatic cancer (9%), liver cancer (18%), and esophageal cancer (19%) [5].

Lung cancer occurs in the respiratory epithelium and is largely pathologically divided into small cell cancer and non-small-cell cancer. Non-small-cell cancer is also classified into squamous cell carcinoma, adenocarcinoma, and large cell carcinoma, divided into 35%, 27%, and 10% of all lung cancer [6]. In stage I-II lung cancer, the 5-year relative survival rate was 53–92% with surgical therapy; but in inoperable stage III-IV lung cancer, despite combination treatment with radiotherapy and chemotherapy, median survival is about 2 years and the 5-year relative survival rate is only 15–20% [7].

The development of molecular biology has led to the discovery of various gene mutations involved in the pathogenesis of non-small-cell lung cancer (NSCLC), which accounts for 85% of lung cancer. Various targeted therapies have been developed based on various gene mutations, leading to a breakthrough in the treatment of lung cancer [8]. However, these targeted therapies show good treatment efficacy in the early stages of use, but they have clear limitations due to their resistance to drugs and their cost [8, 9]. Immunotherapy and adjuvant therapy with natural compounds may be an alternative [8].

Traditional medical treatment has a significant effect in suppressing the growth of cancer cells, reducing the side effects of chemotherapy, and improving the quality of life through single treatment or combined with standard treatments [10, 11]. Therefore, a study to derive an effective traditional medicine for cancer is continuously being conducted [12, 13].

HangAmDan-B1 (HAD-B1) is a prescription consisting of 4 herbal medicines: *Panax notoginseng* (Burk) F. H. Chen, *Cordyceps militaris*, *Panax ginseng* C.A. Meyer, and *Boswellia carterii* Birdwood [14, 15]. HAD-B1 has been reported to have an anti-lung-cancer effect by cell proliferation inhibition in lung cancer xenograft animal experiments using A549 cells and to have an effect of inducing apoptosis and arresting the cell cycle in A549 cisplatin/resistance cells [14, 16]. Therefore, it was considered desirable to conduct safety pharmacology tests in the nonclinical study in order to prescribe HAD-B1 to patients or to conduct clinical trials.

Safety pharmacology tests in accordance with ICH guidelines are increasingly required for the development and clinical trial of new drugs [17]. The ICH guideline S7A defines safety tests for essential life-support organs, the central nervous system, the respiratory system, and the cardiovascular system as core battery tests [18]. In addition, S7B requires in vitro tests, such as hERG assay, or nonclinical tests in vivo to identify the possibility of prolonging the QT interval of drugs and to identify the risk of arrhythmia [19]. Therefore, based on the ICH guideline S7A, the effect on the respiratory system and central nervous system after single oral administration of HAD-B1 in Sprague-Dawley rats was evaluated and the effect on the cardiovascular system was evaluated in beagle dogs using the telemetry system. In addition, the hERG assay for HAD-B1 in CHO hERG cells was performed to confirm the possibility of inducing the QT interval prolongation by HAD-B1 based on S7B guidelines.

## 2. Materials and Methods

### 2.1. Overview

This safety pharmacology test was conducted on the basis of ICH guidelines S7A and S7B, and all animal tests were conducted in accordance with the Guide for the Care and Use of Laboratory Animal by ILAR Publication.

Two HAD-B1 single oral administration tests in rats and hERG cell assay were conducted by the Korea Institute of Toxicology (Daejeon, South Korea), and a single oral administration test in beagle dogs using the telemetry system was conducted by ChemOn Inc. (Yongin, South Korea).

The Korea Institute of Toxicology obtained AAALAC International (Association for Assessment and Accreditation of Laboratory Animal Care International) certification in 1998, and ChemOn obtained AAALAC International certification in 2010. All animal tests passed the IACUC (Institute Animal Care and Use Committee) review.

### 2.2. HAD-B1 Extract

The test article HAD-B1 used in this study consists of 4 herbs (Table 1). The 3D HPLC analysis of HAD-B1 showed the representative 6 critical compounds including notoginsenoside R1, cordycepin, ginsenoside Rg1, Rb1, *α*-boswellic acid, and *β*-boswellic acid [20]. HAD-B1 was supplied by KyungbangPharm Inc. (Incheon, South Korea). The HAD-B1 was shaded and stable for 8 days when stored in a refrigerator and stable for 24 hours when stored at room temperature. HAD-B1 was collected and analyzed for the upper, middle, and lower, and the coefficients of variation (CVs) of the top, middle, and bottom were within 10% and judged to be homogeneous (200 mg/mL: 0.5%). The average content was within the acceptance range within ±15% of the prescribed dose (200 mg/mL: 91.1%).

### 2.3. Evaluation of the Effect on the Cerebral Nerve System in Rats

#### 2.3.1. Animals

70 (35 males and 35 females) SD rats (Orientbio Inc., Seongnam, South Korea) of specific pathogen-free (SPF) of about 4 weeks old at the time of acquisition were used. SD rats were selected because they are widely used for the safety assessment of drugs and have many basic data to be compared [21]. The acclimation period was 9 days, and the rats were quarantined and acclimated in the experimental environment for a period of 4–7 days before administration. General symptoms including mortality, morbidity, appearance, and behavioral changes were observed once a day during the acclimation period to confirm that there was no abnormality. The body weight of the rats was measured once each at the time of acquisition, group separation, and administration period. At the end of the acclimation period, 32 males and 32 females, which showed no clinical symptoms such as disease or wounds and showed appropriate body weight (males: 208.0–253.9 g, females: 154.7–189.1 g), were selected and used for the test.

#### 2.3.2. Dosage and Administration

Based on the most recently measured body weight, 8 males and females per group were assigned to each using Pristima System V7.2 (Xybion Medical System Co., USA). Each animal was randomly placed in the test group (T1, T2, T3) and the control group. The dose of HAD-B1 was determined based on the results of the previous 13-week repeated oral administration toxicity test in rats [20]. No toxicological effects were observed for HAD-B1 up to 2000 mg/kg, thereby determining the no observable adverse effect level (NOAEL) of HAD-B1 to 2000 mg/kg. Based on this result, the high dose in this test was set to 2000 mg/kg (T3), and the medium dose and low dose were set to 1000 (T2) and 500 mg/kg (T1), respectively, with a common ratio of 2. The control group was set to administer an excipient (sterile distilled water). The dose was calculated according to the dose of each group based on the most recently measured body weight, and the administered test substance was continuously stirred using a magnetic stirrer before and during administration. HAD-B1 and the control substance were administered orally once at 10 : 30–11 : 00 a.m. after the animals to be administered were fasted for at least 3 hours before administration.

#### 2.3.3. Observations and Measurement

In order to evaluate the effect on the CNS, neurobehavior was observed and body temperature was measured. General symptoms including mortality, morbidity, changes in appearance, and behavior were observed once a day during the acclimatization period, before administration, and during the administration period and recorded together with the observation date and time. Neurobehavior observations were performed with the modified Irwin's test and open test and were observed at 0 (before), 1, 2, 4, 6, and 24 hours (±10%) after administration. The degree and judgment of the observation items were carried out in accordance with the standard operating procedure. During general behavior observation, rectal temperature was measured simultaneously. After the end of the experiment, all animals used in the test were euthanized with CO_2_.

#### 2.3.4. Statistical Analysis

All values were expressed as mean ± standard deviation (SD). SAS/STAT, version 9.2 (SAS Institute Inc., USA), was used for statistical analysis of the data. Multiple comparison analysis was performed for group comparison. All the data were tested for equality of variance using the Bartlett Test and were analyzed with the one-way ANOVA and Kruskal–Wallis test. The difference between the groups was analyzed by Dunnett's test and Dunn's rank sum test.

### 2.4. Evaluation of the Effect on the Respiratory System in Rats

#### 2.4.1. Animals

35 male SD rats (Orientbio Inc., Seongnam, South Korea) of SPF of about 5 weeks old at the time of acquisition were used. The acclimation period was 3 days, and the rats were quarantined and acclimated in the experimental environment for a period of 3–6 days before administration. General symptoms including mortality, morbidity, appearance, and behavioral changes were observed once a day during the acclimation period to confirm that there was no abnormality. The body weight of the rats was measured once each at the time of acquisition, group separation, and administration period. At the end of the acclimation period, 32 rats, which showed no clinical symptoms such as disease or wounds and showed appropriate body weight, were selected and used for the test. Based on the most recently measured body weight, 8 rats per group were assigned to each using the Pristima System (version 7.2; Xybion Medical System Co., USA). Each animal was randomly placed in the test group (T1, T2, T3) and the control group.

#### 2.4.2. Dosage and Administration

The dose of HAD-B1 was set to 500 mg/kg (T1), 1000 mg/kg (T2), and 2000 mg/kg (T3) as in evaluating the effect on the CNS in rats, and an excipient (sterile distilled water) was administered to the control group. The dose was calculated according to the dose of each group by referring to the most recently measured body weight. HAD-B1 and the control substance were administered orally once.

#### 2.4.3. Observations and Measurement

The evaluation of the effect on the respiratory system was made by measuring the respiratory rate, tidal volume, and minute volume using a respiration analyzer (Unrestrained whole body plethysmograph system; BUXCO Electronics Inc., USA). Measurements were performed according to the standard operating procedure. Measurements were made at 0 (before), 1, 2, 4, 6, and 24 hours (±10%) after administration. Data were collected and stored using BioSystem (BUXCO Electronics Inc., USA). After the end of the experiment, all animals used in the test were euthanized with CO_2_.

#### 2.4.4. Statistical Analysis

All values were expressed as mean ± SD. SAS/STAT, version 9.2, was used for statistical analysis of the data. Multiple comparison analysis was performed for group comparison. All the data were tested for equality of variance using the Bartlett Test and were analyzed with the one-way ANOVA and Kruskal–Wallis test. The difference between the groups was analyzed by Dunnett's test and Dunn's rank sum test.

### 2.5. hERG Assay

The hERG assay was conducted using 12 CHO (Chinese hamster ovary) hERG cells (bSys GmbH, Switzerland). The cells in the liquid nitrogen tank were thawed and cultured for at least 7 days before being used and subcultured at least twice a week in an incubator at 37°C containing about 5% carbon dioxide and saturated water vapor.

HAD-B1 at a predetermined concentration for each group was prepared on the day of measurement by adding a normal tyrode solution to the original HAD-B1 solution, taking a solution from the intermediate layer while stirring, and diluting it in a normal tyrode solution. The excipient was prepared by diluting the normal tyrode stock solution 10 times with tertiary distilled water on the day of measurement. E-4031, which acts as a selective inhibitor on the hERG potassium channel, a voltage-gated potassium channel expressed in the heart, was used as a positive control.

E-4031 was dissolved in distilled water and prepared as a 100 *μ*M stock solution, then dispensed into 0.1 mL, and stored frozen. Frozen 0.1 ml of 100 *μ*M E-4031 was thawed one by one on the day of the experiment, diluted in normal tyrode solution, and then a final concentration of 100 nM E-4031 was prepared.

HAD-B1 was found to inhibit −0.4% and 2.3% hERG potassium channel current at concentrations of 0.1 *μ*g/mL and 1 *μ*g/mL, respectively, in previous tests. Due to the solubility limit of HAD-B1, the highest concentration was set at 1 *μ*g/mL, and the lower concentration group was set on a log scale. The negative control group was set to the normal tyrode solution, test group 1 (T1) was set to 0.1 *μ*g/ml, test group 2 (T2) was set to 0.3 *μ*g/ml, and test group 3 (T3) was set to 1 *μ*g/m. And three cells were used for each group. As the positive control group, random cells were selected from the negative control group, T1, T2, and T3 without any separate cell assignment, and after the measurement was completed, additional E-4031 was perfused.

Electrophysiological measurements were made as follows. After sinking the cells into the chamber, perfuse the normal tyrode solution, confirm that the hERG potassium channel is constantly recorded for at least 3 minutes, and then perfuse each test substance for at least 5 minutes, and then recording was ended after confirming that the size of the hERG potassium channel was recorded consistently.

The microcurrent through the cell membrane was recorded using the whole-cell voltage clamp method. The glass electrode was made of borosilicate glass, and a microelectrode puller was used for fabrication. The current was amplified and digitized using an Axopatch 200B amplifier (Molecular Devices, USA), and the obtained data were stored and analyzed using NOTOCORD Systems, version 4.2 (Croissy-sur-Seine, France). All experiments were performed at 36.8–37.1°C using a dual automatic temperature controller. Pulse protocol overpolarized from holding potential −80 mV to −90 mV for 100 ms, depolarized to +20 mV for 2 s, and then repolarized again to −40 mV for 3 s to create tail currents continuously pulses at intervals of about 20 seconds. After the stimulation of the control, the test substance solution was perfused and further stimulation continued. The peak of the tail current was measured and analyzed, and the pulse frequency was 0.05 Hz. After recording the magnitude of the current before and after perfusion of the test substance by the NOTOCORD-hem program, it was transferred to Excel 2003 (Microsoft, USA), and the average value of data for 1 minute (3 points) was used as the data at the corresponding concentration. The leak current was checked before the test pulse, and the size of the leak current was combined with the measured tail current to obtain the total hERG potassium channel current size. The relative current size and suppression rate were obtained by substituting the current before and after administration for the following formula. All figures were calculated by rounding them from the second decimal place.

(1) $$\begin{array}{c} \text{relative current}=\frac{\text{current after perfusion }}{\text{current before perfusion }},\\ \text{suppression rate }\left(\%\right)=\left(1-\text{relative current}\right)\times 100. \end{array}$$

Results were expressed as mean ± standard mean error (SEM). Multiple comparison analysis was performed for group comparison. All the data were tested for equality of variance using the Bartlett Test and were analyzed with one-way ANOVA. The difference between the groups was analyzed by Dunnett's test. Between the negative and positive controls, Student's *t*-test, which is used to test the significance between the two groups, was performed. All tests were tested with significance levels *p* < 0.05 and *p* < 0.01.

### 2.6. Evaluation of the Effect on the Cardiovascular System in Beagle Dogs

#### 2.6.1. Animals

6 male beagle dogs (Beijing Marshall Biotechnology Co. Ltd, China) of about 24–43.5 months of age at the time of acquisition were selected and acclimatized for 7 days until the day before administration. After the end of the acclimatization period, 4 animals, which show no abnormality in hematology and blood biochemical test results and cardiovascular index (blood pressure, heart rate, electrocardiogram), were finally selected and used for the test. General symptoms were observed once a day during the acclimation period, and the cardiovascular index was checked using a telemetry system at the group separation.

#### 2.6.2. Dosage and Administration

In the previous test, as a result of repeated oral administration of HAD-B1 to beagle dogs at a dose of 2000 mg/kg/day for 4 weeks, the effect of HAD-B1 was not observed, so the maximum dose was determined to be 2000 mg/kg (G4). The control group (G1) was administered an excipient, 500 mg/kg was administered to the G2 group, and 1000 mg/kg was administered to the G3 group. The administration was carried out by oral administration, which is the clinical route, and the test substance for each dose was administered using a gelatine capsule for oral administration. The dose was calculated based on the body weight measured after fasting on the day of administration. The excipient and the test substances were administered to each animal a total of 4 times, once for each dose. After one administration, a wash-out period of 7 days was set and administered according to the schedule shown in Table 2.

#### 2.6.3. Observations and Measurement

Observation of general symptoms was recorded by visual observation once a day on the day of administration (day 1) and the day after administration (day 2). In addition, observation was continued until 1 hour after administration and every hour until 6 hours through a monitor connected to a video camera on day 1.

The cardiovascular system was evaluated using the Ponemah™ (DSI, USA) telemetry system. It was confirmed that blood pressure, heart rate, electrocardiogram (ECG), and body temperature were stabilized before administration of the test substance, and up to 24 hours after administration, heart rate, systolic blood pressure, diastolic blood pressure, mean blood pressure, body temperature, and Lead II ECG (PR , QRS , QT , RR , and QTc intervals) were continuously collected. After data collection was completed, the ECG waveform could be read at each measurement time (before administration, 1, 2, 4, 6, 24 hours (±10%) after administration). After selecting data for 1 minute with stable signal, Excel (Microsoft, USA) files were extracted and used as test basic data. Ten consecutive ECG waveforms were selected at each measurement time and used for waveform diagnosis.

Comparisons were made between groups through parametric multiple comparison procedures. For heart rate, systolic blood pressure, diastolic blood pressure, mean blood pressure, body temperature, and ECG, the one-way ANOVA test was applied to the comparison between the vehicle control group and the test substance administration group. The significance level was *p* < 0.05.

## 3. Results

### 3.1. Evaluation of the Effect on the CNS in Rats

No abnormal neurobehavior was observed in all male and female rats administered with the excipient and HAD-B1.

As a result of body temperature measurement, in males administered with HAD-B1, no significant change in body temperature was observed at all measurement times compared with the vehicle control group. A statistically significant decrease in body temperature was observed in the 1000 mg/kg administration group (38.7 ± 0.2°C) 2 hours after administration compared with the excipient control group (38.9 ± 0.2°C) in females (Table 3).

### 3.2. Evaluation of the Effect on the Respiratory System in Rats

No significant changes were observed in the respiratory rate, tidal volume, and minute volume at all measurement times of all doses (Tables 4–6).

### 3.3. hERG Assay

The excipient inhibited the hERG potassium channel by about 0.9%. HAD-B1 inhibited the hERG potassium channel by about 0.7%, 1.2%, and 3.6%, respectively, at concentrations of 0.1 *μ*g/mL, 0.3 *μ*g/mL, and 1 *μ*g/mL. None of these results were statistically significant.

**Figure 1:**
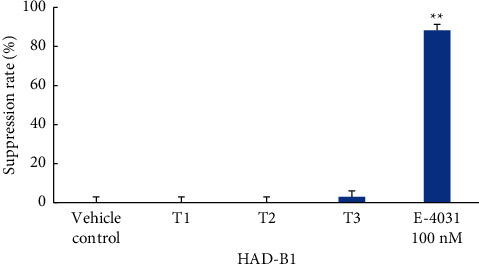
Effects of vehicle control, HAD-B1, and E-4031 on cloned hERG potassium channels expressed in CHO cells. The percentage of current suppression in each group of 3 cells is shown in the bar graph. Values are means, and error bars indicated SEM (standard error of the mean). ^∗∗^Significance at *P* < 0.01, vehicle control vs. E4031, Student's *t*-test.

E-4031 inhibited the hERG potassium channel by about 88.3%, which was statistically significant (*p* < 0.01) (Figure 1).

### 3.4. Evaluation of the Effect on the Cardiovascular System in Beagle Dogs

As a result of observing general symptoms, vomiting was observed 4 hours after administration in the 500 mg/kg group. In the 1000 mg/kg group, one case of feed was observed on day 2. Vomiting was observed 4 hours after administration in the 2000 mg/kg group (Table 7).

**Figure 2:**
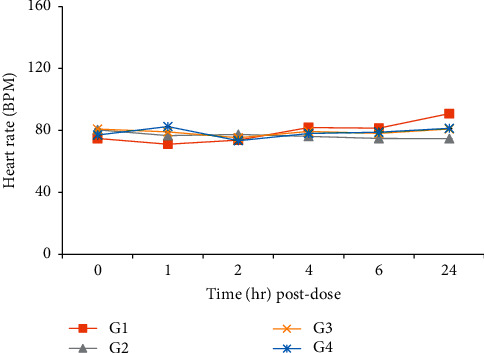
Effects of HAD-B1 on the heart rate in beagle dogs. G1 (HAD-B1, 0 mg/kg), G2 (HAD-B1, 500 mg/kg), G3 (HAD-B1, 1000 mg/kg), G4 (HAD-B1, 2000 mg/kg).

**Figure 3:**
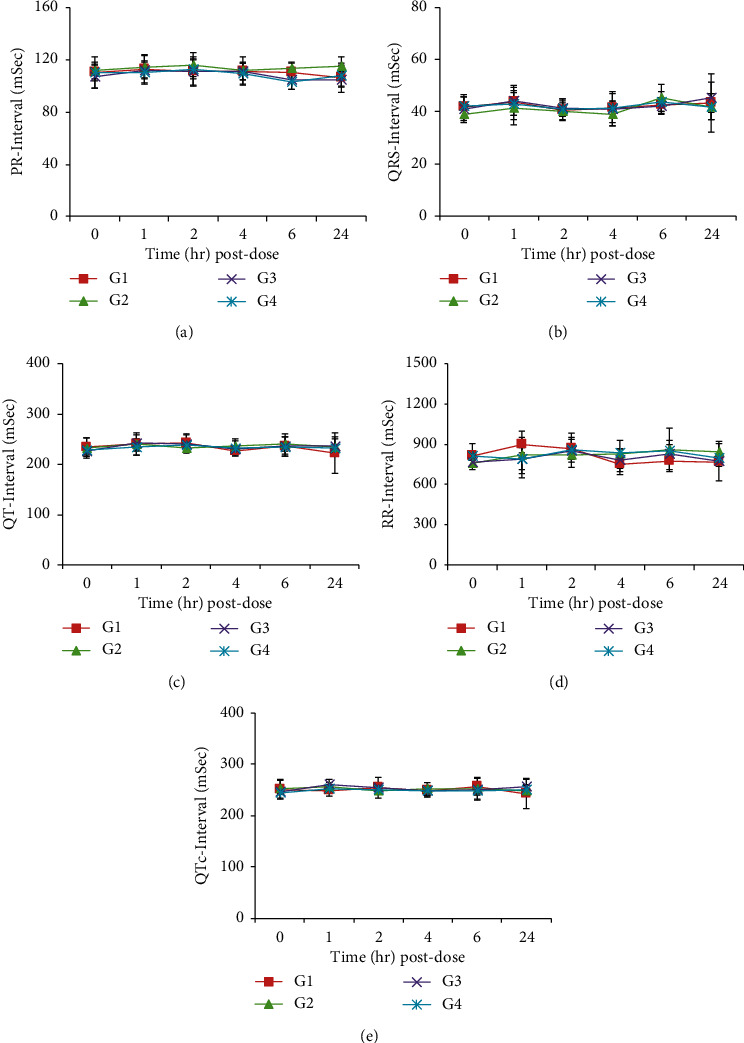
Effects of HAD-B1 on ECG in beagle dogs. G1 (HAD-B1, 0 mg/kg), G2 (HAD-B1, 500 mg/kg), G3 (HAD-B1, 1000 mg/kg), G4 (HAD-B1, 2000 mg/kg). (a) PR interval. (b) QRS interval. (c) QT interval. (d) RR interval. (e) QTc interval.

**Figure 4:**
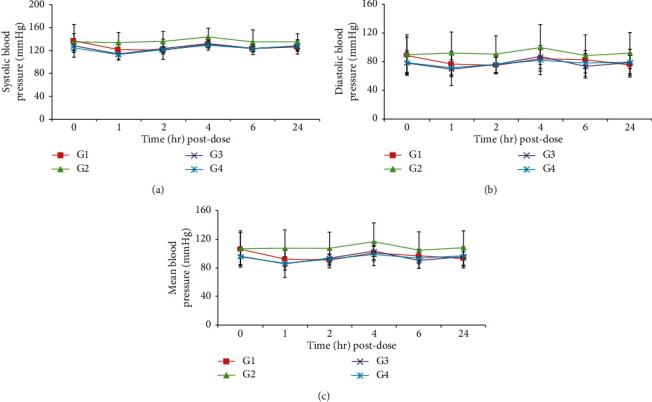
Effects of HAD-B1 on blood pressure in beagle dogs. G1 (HAD-B1, 0 mg/kg), G2 (HAD-B1, 500 mg/kg), G3 (HAD-B1, 1000 mg/kg), G4 (HAD-B1, 2000 mg/kg). (a) Systolic blood pressure. (b) Diastolic blood pressure. (c) Mean blood pressure.

No changes related to the test substance were observed in blood pressure, heart rate, ECG, and body temperature (Figures 2–4, Table 8).

## 4. Discussion

HAD-B1 is a prescription developed by adjusting the dose except for some drugs in HAD-B, and it has been found to have anti-lung-cancer effects as a mechanism to inhibit cell proliferation, induce apoptosis, and arrest the cell cycle in cell tests [14, 16].

In the development of new drugs, nonclinical stability pharmacology and toxicology studies assess the potential risks posed by drugs prior to use in humans. Serious adverse effects associated with the drug should be tested, the potential risk should be evaluated accordingly, and the effectiveness of the drug should be compared. Stability pharmacology and toxicology studies should also be performed to assess these significant potential risks in the development of new drugs using Korean medicines [22, 23].

Representative nonclinical pharmacology stability is a central nervous system test, a respiratory system test, and a cardiovascular test. The assessment of the effects of these three is useful in assessing the lethal risk of a drug. Nerve damage and neurotoxicity of the central nervous system are performed in vivo in rodents using the Irwin test or exercise activity. Cardiovascular testing includes assessment of the effect of appropriate cellular systems on ionic currents, action potential parameters in vitro or in vivo, and ECG measurements in appropriate animal models in accordance with ICH regulations. Some of the tests of respiratory function can be tested in lung cell culture. In addition, respiration rate, oxygen saturation, and arterial blood gas monitoring are also ways to monitor respiratory function.

These central nervous system, respiratory system, and cardiovascular stability tests can be evaluated using pathological methods of each tissue in general toxicity tests, so it is necessary to compare them with general toxicity tests.

Assessing toxicity and understanding the mechanisms in natural product research can help assess potential risk and classify patients for indications in the clinic. In particular, in the case of cancer patients, it causes serious side effects due to toxicity from chemical anticancer agents, so safe drugs are good in terms of such safety and pharmacology.

Therefore, this study was conducted to evaluate the safety of HAD-B1 in the CNS, respiratory system, and cardiovascular system in vivo and in vitro and to evaluate the possibility of prolonging the QT interval related to the risk of arrhythmia. This result was intended to be used as basic data on the safety of HAD-B1, which has anti-lung-cancer effects.

First, to evaluate the effect of HAD-B1 on the CNS, after single oral administration of HAD-B1 at a dose of 0 (excipient), 500, 1000, and 2000 mg/kg to 64 male and female rats, neurobehavior and body temperature was measured at 0, 1, 2, 4, 6, and 24 hours after administration. No abnormal neurobehavior was observed by all doses of the test substances. No significant changes in body temperature were observed in males. A statistically significant decrease in body temperature was observed in females in the 1000 mg/kg group compared with the excipient group 2 hours after administration, but the difference in body temperature between the two groups was about 0.2°C. In addition, the average body temperature (38.7 ± 0.2°C) and individual body temperature (38.3–38.8°C) were in the normal range when compared with historical control data (38.6 ± 0.4°C). Therefore, it is judged that there is no change in body temperature related to the test substance at all measurement times. HAD-B1, based on the above results, does not affect the central nervous system when administered orally at a dose of 2000 mg/kg in male and female rats once.

Second, to evaluate the effect of HAD-B1 on the respiratory system of rats, after single oral administration of HAD-B1 at a dose of 0 (excipient), 500, 1000, and 2000 mg/kg to 32 male rats, respiration rate, tidal volume, and minute volume were measured before administration (0 hours) and at 1, 2, 4, 6, and 24 hours after administration of the test substance. No changes were observed in the respiration rate, tidal volume, and minute volume related to the test substance at all measurement times. HAD-B1, based on this result, does not affect the respiratory system when administered orally at a dose of 2000 mg/kg.

Third, hERG assay was performed to determine the possibility of HAD-B1 inducing a QT interval prolongation. CHO hERG cells were used to evaluate the effect on the hERG potassium channel. As a result of the hERG assay, the hERG potassium channel was inhabited by about 0.9% when the excipient control was perfused. When HAD-B1 was perfused at concentrations of 0.1 *μ*g/mL, 0.3 *μ*g/mL, and 1 *μ*g/mL, about 0.7%, 1.2%, and 3.6% were inhibited, and no statistical significance was shown in excipient and all HAD-B1 groups. When perfused with 100 nM of E-4031, a positive control material, the hERG potassium channel was inhibited by 88.3%, thus demonstrating that this test composition is effective (*p* < 0.01). HAD-B1 has no effect on the hERG potassium channel up to 1 *μ*g/mL.

Finally, to evaluate the effect on the cardiovascular system, HAD-B1 500, 1000, and 2000 mg/kg were administered to male beagle dogs, and the effects on blood pressure, heart rate, ECG, and body temperature were observed using a telemetry system. Vomiting was observed, in general symptoms, 4 hours after administration in the 500 mg/kg and 2000 mg/kg administration groups. In the case of vomiting, however, due to the developed vomiting center in dogs, it is caused by mild gastrointestinal irritation without toxic effects [24]. Therefore, it is determined that the symptoms are not caused by the test substance. No changes related to the test substance were observed as a result of blood pressure and heart rate, ECG, and body temperature observations. Based on the above results, HAD-B1 is judged to have no effect on the cardiovascular system up to 2000 mg/kg dose when oral administration to the beagle dog.

As a result of these safety pharmacology tests, HAD-B1 is considered to be a safe drug in the CNS, respiratory system, and cardiovascular system up to a dose of 2000 mg/kg. These results could be the basis for securing safety in clinical trials and patient administration for HAD-B1.

## 5. Conclusion

As a result of the single oral administration of HAD-B1 in rats and beagle dogs, no effect was found on the CNS, respiratory system, and cardiovascular system up to 2000 mg/kg. As a result of the hERG assay of HAD-B1, the possibility of inducing the QT interval prolongation to 1 *μ*g/mL was not observed. HAD-B1 was identified as a safe drug in preclinical trials following the guidelines of ICH S7A and S7B.

## Figures and Tables

**Table 1:** Ingredients of HAD-B1.

Scientific name	Plant parts used	Geographic location of growth	Representative component	Relative amount (g) (%)
*Panax notoginseng* (Burk) F. H. Chen	Root	China	Notoginsenoside R1	25.2 (32.3)
*Cordyceps militaris*	Fruiting body and corpus	Korea	Cordycepin	19.2 (24.6)
*Panax ginseng* C.A. Meyer	Root	Korea	Ginsenoside Rg1, Rb1	19.2 (24.6)
*Boswellia carterii* Birdwood	Mastic	China	*α*-Boswellic acid, *β*-boswellic acid	14.4 (18.5)
Total amount				78.0 (100)

**Table 2:** Administration cycle for each beagle dog.

No. of animals	Administration cycle			
	1	2	3	4
1	G1	G2	G3	G4
2	G2	G3	G4	G1
3	G3	G4	G1	G2
4	G4	G1	G2	G3

G1: vehicle control group, G2–G4: test article group.

**Table 3:** Effects of HAD-B1 on body temperature in rats.

Sex	Groups	Dose (mg/kg)	Time (hr)					
			0	1	2	4	6	24
Males	Control	0	38.2 ± 0.1	38.3 ± 0.2	38.0 ± 0.2	37.7 ± 0.2	37.6 ± 0.2	38.1 ± 0.2
	T1	500	38.3 ± 0.3	38.3 ± 0.3	38.0 ± 0.3	37.8 ± 0.3	37.6 ± 0.2	38.2 ± 0.3
	T2	1000	38.3 ± 0.3	38.3 ± 0.4	38.0 ± 0.4	37.8 ± 0.2	37.6 ± 0.2	38.1 ± 0.3
	T3	2000	38.3 ± 0.2	38.1 ± 0.2	37.8 ± 0.1	37.7 ± 0.2	37.6 ± 0.2	38.0 ± 0.2

Females	Control	0	38.8 ± 0.3	39.0 ± 0.2	38.9 ± 0.2	38.2 ± 0.2	37.9 ± 0.2	38.7 ± 0.2
	T1	500	39.0 ± 0.3	39.0 ± 0.2	38.9 ± 0.3	38.2 ± 0.3	37.9 ± 0.2	38.8 ± 0.3
	T2	1000	38.9 ± 0.4	38.8 ± 0.2	38.7 ± 0.2^+^	38.0 ± 0.2	37.8 ± 0.1	38.6 ± 0.4
	T3	2000	38.9 ± 0.2	38.9 ± 0.2	38.7 ± 0.2	38.0 ± 0.2	37.8 ± 0.2	38.8 ± 0.3

*Note*. Data described as the mean ± S.D. (*n* = 8).

**Table 4:** Effects of HAD-B1 on respiratory rate in rats.

Groups	Dose (mg/kg)	Time (hr)					
		0	1	2	4	6	24
Control	0	155.56 ± 55.73	125.67 ± 14.02	126.43 ± 8.28	126.47 ± 6.28	149.68 ± 68.76	132.99 ± 7.04
T1	500	141.64 ± 42.61	126.89 ± 12.44	130.36 ± 11.82	124.55 ± 10.93	127.97 ± 15.24	160.23 ± 58.23
T2	1000	134.15 ± 11.60	143.62 ± 30.75	143.15 ± 41.28	127.56 ± 17.29	151.21 ± 92.61	158.51 ± 37.51
T3	2000	150.02 ± 39.22	140.23 ± 13.25	167.29 ± 56.98	129.05 ± 14.71	147.20 ± 32.46	141.01 ± 23.54

*Note*. Data described as the mean ± S.D. (*n* = 8).

**Table 5:** Effects of HAD-B1 on tidal volume (mL) in rats.

Groups	Dose (mg/kg)	Time (hr)					
		0	1	2	4	6	24
Control	0	1.23 ± 0.18	1.20 ± 0.15	1.16 ± 0.24	1.15 ± 0.13	1.17 ± 0.20	1.25 ± 0.17
T1	500	1.24 ± 0.07	1.19 ± 0.09	1.14 ± 0.11	1.12 ± 0.07	1.14 ± 0.12	1.27 ± 0.16
T2	1000	1.22 ± 0.17	1.21 ± 0.16	1.18 ± 0.12	1.14 ± 0.11	1.21 ± 0.18	1.34 ± 0.19
T3	2000	1.15 ± 0.14	1.15 ± 0.08	1.13 ± 0.14	1.14 ± 0.20	1.16 ± 0.16	1.26 ± 0.15

*Note*. Data described as the mean ± S.D. (*n* = 8).

**Table 6:** Effects of HAD-B1 on minute volume in rats.

Groups	Dose (mg/kg)	Time (hr)					
		0	1	2	4	6	24
Control	0	183.44l38.67	147.86l19.95	144.05l29.36	142.91l15.68	171.22l82.51	164.56l20.34
T1	500	174.77l60.05	148.36 ± 18.94	144.65 ± 18.9	136.74 ± 18.94	142.38 ± 18.94	201.24 ± 18.94
T2	1000	160.41 ± 18.94	170.56 ± 18.94	166.32 ± 18.94	140.82 ± 18.9	173.41 ± 18.94	211.70 ± 18.94
T3	2000	170.06 ± 18.94	157.98 ± 18.9	190.25 ± 18.94	142.61 ± 18.94	165.82 ± 18.94	176.01 ± 18.94

*Note*. Data described as the mean ± S.D. (*n* = 8).

**Table 7:** Effects of HAD-B1 on clinical signs in beagle dogs.

Days	Signs	Groups (mg/kg)			
		G1 (0)	G2 (500)	G3 (1000)	G4 (2000)
1	Normal	4/4	3/4	4/4	3/4
	Vomiting	0/4	1/4	0/4	1/4

2	Normal	4/4	4/4	3/4	4/4
	Remaining of food	0/4	0/4	1/4	0/4

**Table 8:** The number of animals showed abnormal ECG waveform.

Groups	Dose (mg/kg)	Time (hr)					
		0	1	2	4	6	24
G1	0	0	0	0	0	0	0
G2	500	0	0	0	0	0	0
G3	1000	0	0	0	0	0	0
G4	2000	0	0	0	0	0	0

## Data Availability

The data used to support the findings of this study are included within the article.
